# Downregulation of Nitric Oxide Collaborated with Radiotherapy to Promote Anti-Tumor Immune Response via Inducing CD8+ T Cell Infiltration

**DOI:** 10.7150/ijbs.41653

**Published:** 2020-03-05

**Authors:** Jieyu Xu, Yuan Luo, Cheng Yuan, Linzhi Han, Qiuji Wu, Liexi Xu, Yuke Gao, Yingming Sun, Shijing Ma, Guiliang Tang, Shuying Li, Wenjie Sun, Yan Gong, Conghua Xie

**Affiliations:** 1 Department of Radiation and Medical Oncology, Zhongnan Hospital of Wuhan University, Wuhan, China; 2 Department of Biological Repositories, Zhongnan Hospital of Wuhan University, Wuhan, China; 3 Hubei Key Laboratory of Tumour Biological Behaviors, Zhongnan Hospital of Wuhan University, Wuhan, China; 4 Hubei Cancer Clinical Study Center, Zhongnan Hospital of Wuhan University, Wuhan, China

**Keywords:** iNOS, myeloid cells, radiotherapy, immunotherapy, tumor microenvironment

## Abstract

The production of nitric oxide (NO) is a key feature of immunosuppressive myeloid cells, which impair T cell activation and proliferation via reversibly blocking interleukin-2 receptor signaling. NO is mainly produced from L-arginine by inducible NO synthase (iNOS). Moreover, L-arginine is an essential element for T cell proliferation and behaviors. Impaired T cell function further inhibits anti-tumor immunity and promotes tumor progression. Previous studies indicated that radiotherapy activated anti-tumor immune responses in multiple tumors. However, myeloid-derived cells in the tumor microenvironment may neutralize these responses. We hypothesized that iNOS, as an important regulator of the immunosuppressive effects in myeloid-derived cells, mediated radiation resistance of cancer cells. In this study, we used 1400W dihydrochloride, a potent small-molecule inhibitor of iNOS, to explore the regulatory roles of NO in anti-tumor immunity. Radiotherapy and iNOS inhibition by 1400W collaboratively suppressed tumor growth and increased survival time, as well as increased tumor-infiltrating CD8^+^ T cells and specific inflammatory cytokine levels, in both lung and breast cancer cells *in vivo*. Our results also suggested that myeloid cell-mediated inhibition of T cell proliferation was effectively counteracted by radiation and 1400W-mediated NO blockade *in vitro*. Thus, these results demonstrated that iNOS was an important regulator of radiotherapy-induced antitumor immune responses. The combination of radiotherapy with iNOS blockade might be an effective therapy to improve the response of tumors to clinical radiation.

## Introduction

Immunotherapy plays increasingly important roles during tumor treatment, especially in assisting radiotherapy (RT) to improve clinical outcomes. However, RT has inhibitory effects on immune activation through various mechanisms. Radiation can recruit myeloid-derived suppressive cells that inhibit the activation and function of T cells 1-5. Tumor-infiltrating myeloid-derived cells are mainly composed of dendritic cells, myeloid-derived suppressor cells (MDSCs), polymorphonuclear neutrophils, and tumor-associated macrophages (TAMs) 6-8.

The general characteristic of these cells is the production of nitric oxide (NO) 4, 9-11. The synthesis of NO is catalyzed by NO synthase (NOS), using L-arginine as the substrate 12. There are 3 known isoforms of NOS: neuronal NOS (nNOS), inducible NOS (iNOS), and endothelial NOS (eNOS) 13, of which iNOS is the majority one 14, 15. NO can directly suppress the activation and proliferation of T cells by reversibly intercepting signals through the interleukin (IL)-2 receptor and inducing T cell apoptosis 2-4. iNOS can produce NO and thus deplete L-arginine, which is an essential nutrient for the activation and functional expression of T cells 16-18.

Experimental data indicated that immune responses activated by RT could be impeded by production of NO. L-n6-(1-iminoethyl)-lysine (L-NIL), as a potent iNOS inhibitor, enhanced RT susceptibility of treatment-refractory head and neck cancer 19, 20. Moreover, NO reduction with a highly-selective NOS inhibitor, NG-monomethyl-L-arginine monoacetate (LNMMA), enhanced the radiosensitization of non-small cell lung cancer (NSCLC) cells 21. However, the underlying mechanism was still to be investigated. Therefore, we hypothesized that inhibition of iNOS could effectively alleviate RT-inhibited of immune system activation and promoted T cell function via suppressing NO synthesis in immunosuppressive myeloid cells, thereby further enhanced the efficacy and clinical outcomes of RT.

In the present study, we assessed the efficacy of the small-molecule inhibitor of iNOS, 1400W dihydrochloride, using xenograft mouse models* in vivo*. The results showed that 1400W combined with RT treatment significantly suppressed tumor growth and extended survival time. Immune cells in the tumor microenvironment and spleen tissues were further analyzed to explore the mechanism of the inhibitory effects of iNOS inhibitor. An increased number of tumor-infiltrating CD8^+^ T lymphocytes was detected in the combined treatment group. Similarly, the numbers of CD8^+^ and CD4^+^ T lymphocytes in the spleen increased significantly. We further validated our hypothesis with T cell proliferation assay *in vitro*. Our results indicated that iNOS inhibition combined with RT collaboratively suppressed tumor growth, providing a new strategy for cancer immunotherapy.

## Materials and Methods

### Mice

Female C57BL/6 (6-8 weeks old) and Balb/c mice (5-7 weeks old) were supplied by Vital River Laboratory Animal Technology (Beijing, China) and housed in a specific-pathogen-free environment. All animal experiments were approved by the Institutional Animal Care and Use Committee of Wuhan University. The animals were sacrificed when reached the Mice Welfare Endpoint.

### Cells and reagents

Lewis lung carcinoma (LLC), 4T1, THP-1 and Jurkat cells were purchased from the Cell Resource Center of the Shanghai Institutes for Biological Sciences of the Chinese Academy of Sciences (Shanghai, China). LLC and 4T1 cells were cultured in DMEM (HyClone, Logan, USA) supplemented with 10% fetal bovine serum and 1% glutamine-penicillin-streptomycin at 37 °C in a 10% CO_2_ atmosphere. THP-1 and Jurkat cells were cultured in RPMI-1640 medium (HyClone) supplemented with 15% inactivated fetal bovine serum and 1% glutamine- penicillin-streptomycin at 37°C in a 10% CO_2_ atmosphere. LLC-luc and 4T1-luc cells were generated by luciferase lentivirus (LV-NC, OBiO Technology, Shanghai, China) infection and puromycin (Cayman Chemical, Ann Arbor, USA) selection (3 mg/mL for LLC and 2 mg/mL for 4T1). 1400W dihydrochloride, a potent small-molecule inhibitor of iNOS, was purchased from MedChemExpress (Monmouth Junction, USA).

### Tumor inoculation and treatment

**Figure 1:**
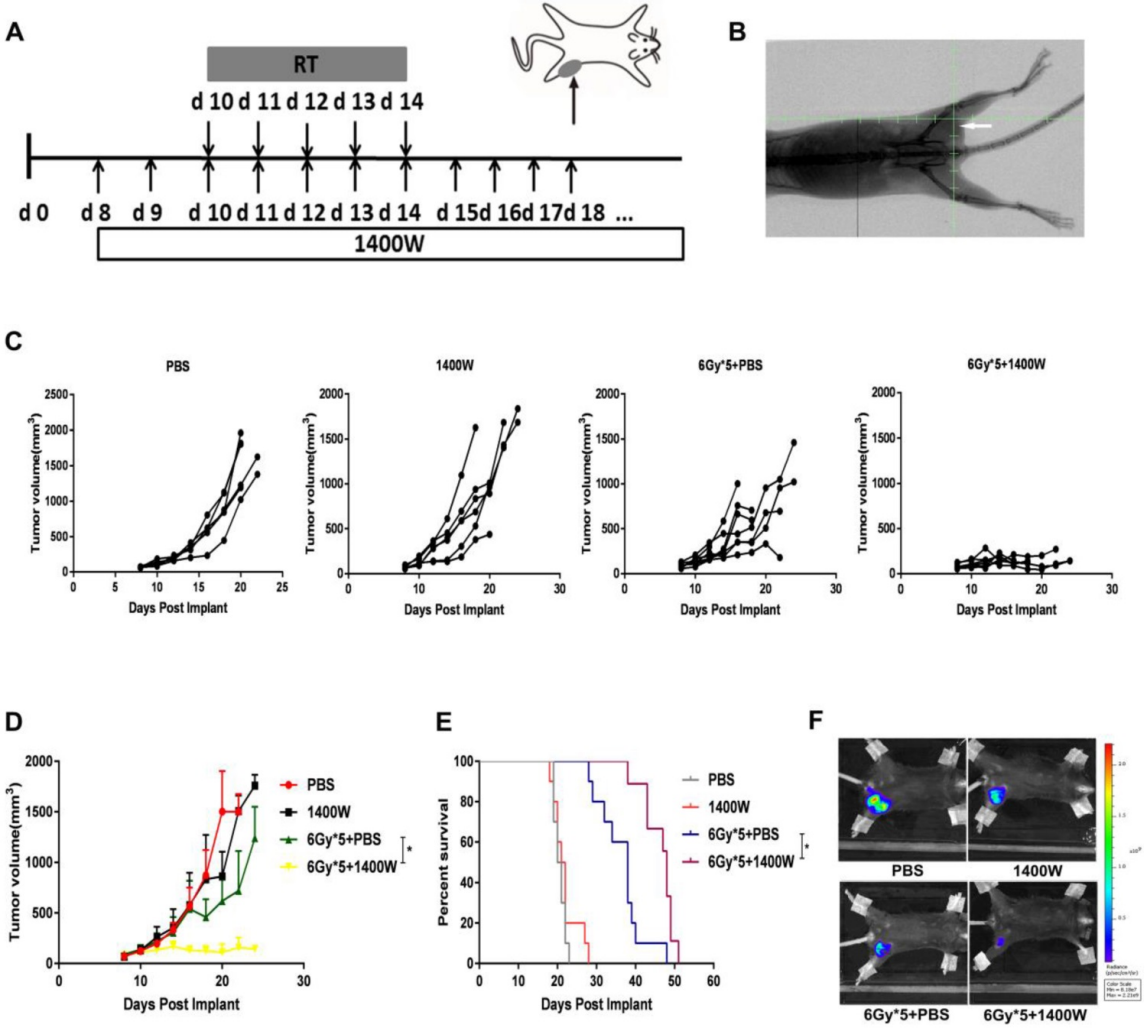
** iNOS inhibition enhanced lung cancer cell radiosensitivity *in vivo*.** (A): Treatment schema of lung cancer xenograft mouse study. (B): Representative X-ray image of positioning verification before irradiation. (C): Individual mouse tumor growth curves. (D): Mean tumor volume of each group. *, *P* < 0.05. (E): Survival curves. PBS (n = 10), 1400W (n = 10), 6Gy × 5 + PBS (n = 10), 6Gy × 5 + 1400W (n = 9). *, *P* < 0.05. (F): Representative *in vivo* images of each group. *, *P* < 0.05.

C57BL/6 and Balb/c mice were injected subcutaneously with 1×10^6^ LLC-luc or 4T1-luc cells in the left lower flank respectively 22. When the tumor size reached approximately 120 mm^3^, mice were randomly grouped: control, radiation, iNOS inhibition, radiation and iNOS inhibition. X-ray fluoroscopy was used to verify the completed foci of the irradiated field and tumors were then irradiated with a dose of 6 Gy × 5, using a small animal irradiation research platform (PXI X-RAD 225Cx, Precision X-Ray, North Branford, USA; Fig. 1A&B). Tumor volume was measured using a Vernier caliper every other day and calculated according to the formula: length × width × width/2. 1400W or vehicle control (phosphate-buffered saline, PBS) were administered intraperitoneally (200 μg/mouse) every day from day 8 for LLC and day 5 for 4T1 23.

For *in vivo* bioluminescence imaging, mice were anesthetized with pentobarbital (Sigma Chemical, St Louis, USA). D-luciferin (PerkinElmer, Waltham, USA) was intraperitoneally injected into the belly, and images were captured using an IVIS Lumina XRMS Series III imaging system (PerkinElmer). CD4^+^ and CD8^+^ T cells were depleted by intraperitoneally injecting 250 mg of monoclonal α-CD4 (GK1.5 clone, BioXCell, West Lebanon, USA) and α-CD8 (2.43 clone, BioXCell) antibodies, and depletion was confirmed by mononuclear cell staining in peripheral blood. Animals were sacrificed according to humane endpoint guidelines: tumors necrotized or reached the size of approximately 2,000 mm^3^.

For survival analysis, each group was stated with 9 to 10 mice. In addition to death, mice were sacrificed when the following points were reached: 1, tumors necrotized or reached the size of approximately 2,000 mm^3^; 2, the tumor influenced breathing, eating, walking and any other physiologic functions; 3, rupture appears on the surface of the tumor. Log-rank tests were used to assess differences in survival.

### Flow cytometry

Single-cell suspensions were generated by tumor excision and collagenase digestion. The cells were then stained with fluorescence-labeled antibodies against CD4 (BD Biosciences, Franklin Lakes, USA), CD8 (BD Biosciences), and CD45 (BD Biosciences). The samples were examined by FACS Aria TM III Cell Sorter (BD Biosciences) and data were analyzed with FlowJo software.

### Serum cytokine analysis

Serum samples were collected by centrifuging peripheral blood at 5,000 rpm for 5 min and then at 3000 rpm for 5 min. The levels (pg/mL) of interleukin (IL)-2, IL-4, IL-5, and interferon (IFN)-γ in the serum samples were assessed using Cytometric Bead Array (CBA) Mouse Th1/Th2 Cytokine Kit (BD Biosciences) according to the manufacturer's instructions. The samples were then analyzed using a FACS Aria TM III Cell sorter and data were analyzed using BD Biosciences CBA software.

### T cell proliferation assay

THP-1 cells were seeded into 6-well plates and induced with phorbol 12-myristate 13-acetate. After incubation for 12 h and observation of adherent growth, the THP-1 cells were randomized into 4 groups treated as follows: blank control, 4 Gy radiation, 1400W treatment (60 μM), 4 Gy radiation combined with 1400W treatment (60 μM) 24. After incubation for 12 h and 24 h, the supernatant was collected and applied to Jurkat cells cultured in 6-well plates. The numbers of viable cells were calculated using a blood cell counting plate 24 h later.

### Statistical analysis

All experiments were performed in triplicates. Results are expressed as mean ± SEM. The unpaired Student t test was used to analyze cytokine levels and cell numbers between groups. Treatment effects on tumor growth were assessed using One-way analysis of variance. Log-rank tests were used to assess differences in survival. *P* values less than 0.05 were considered statistically significant.

## Results

### iNOS inhibition and RT cooperatively suppressed tumor growth

1400W was previously reported to efficiently inhibit iNOS and widely used as iNOS inhibitor 24-26. To determine whether 1400W inhibited tumor growth and enhanced RT efficacy, its effects in both lung and breast cancer cells were examined in the xenograft mouse models. Changes in tumor volume were recorded and tumor growth curves were plotted to investigate the efficacy of 1400W alone or in combination with RT in tumor-bearing mice (Fig. 1C&D). Treatment with 1400 W alone had no substantial effect on tumor growth, but significantly suppressed tumor growth in the combined treatment group compared with the single-fractionated RT group (*P* < 0.05). To further observe whether iNOS inhibition improved the therapeutic effect of RT, survival analysis was performed. Our results indicated that combination therapy effectively prolonged survival time (Fig. 1E). *In vivo* imaging results also demonstrated that 1400W combined with RT collaboratively inhibited tumor progression than RT alone (Fig. 1F). All of the above experimental results indicated the synergistic effects of iNOS inhibition and RT on inhibiting tumor growth.

**Figure 2:**
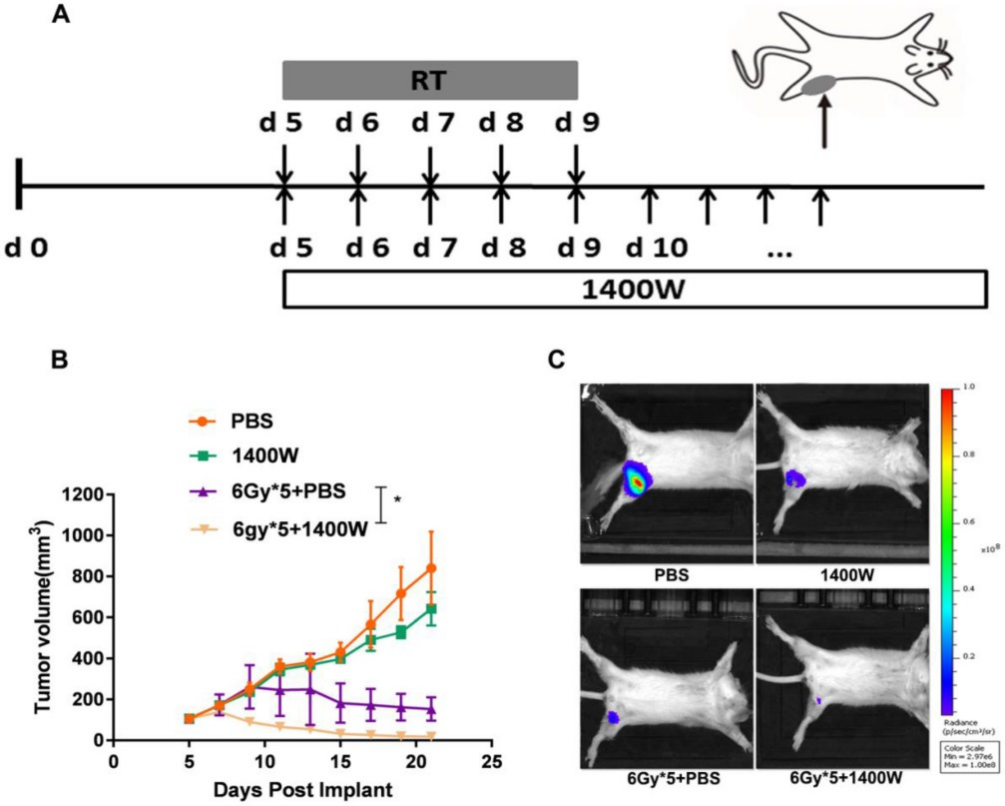
** iNOS inhibition enhanced breast cancer cell radiosensitivity *in vivo.*** (A): Treatment schema of breast cancer xenograft mouse study. (B): Tumor growth curves. *, P < 0.05. (C): Representative *in vivo* images of each group. n = 9-10; *, *P* < 0.05.

To investigate the efficacy of iNOS inhibition in other tumors, a 4T1 tumor model was subsequently established and when the tumors were palpable, mice were randomly assigned to four groups and treated as described above (Fig. 2A). Similar to the LLC model, no significant effect on tumor growth was observed in the group administered 1400W alone. However, iNOS inhibition significantly delayed 4T1 tumor progression when combined with RT (Fig. 2B, *P* < 0.05). *In vivo* imaging of tumor-bearing Balb/c mice also indicated that the combination of 1400W and RT inhibited tumor growth to a greater extent than individual RT treatment in the breast cancer model (Fig. 2C).

### iNOS inhibition potentiated the therapeutic effects of RT in a T cell-dependent manner

**Figure 3:**
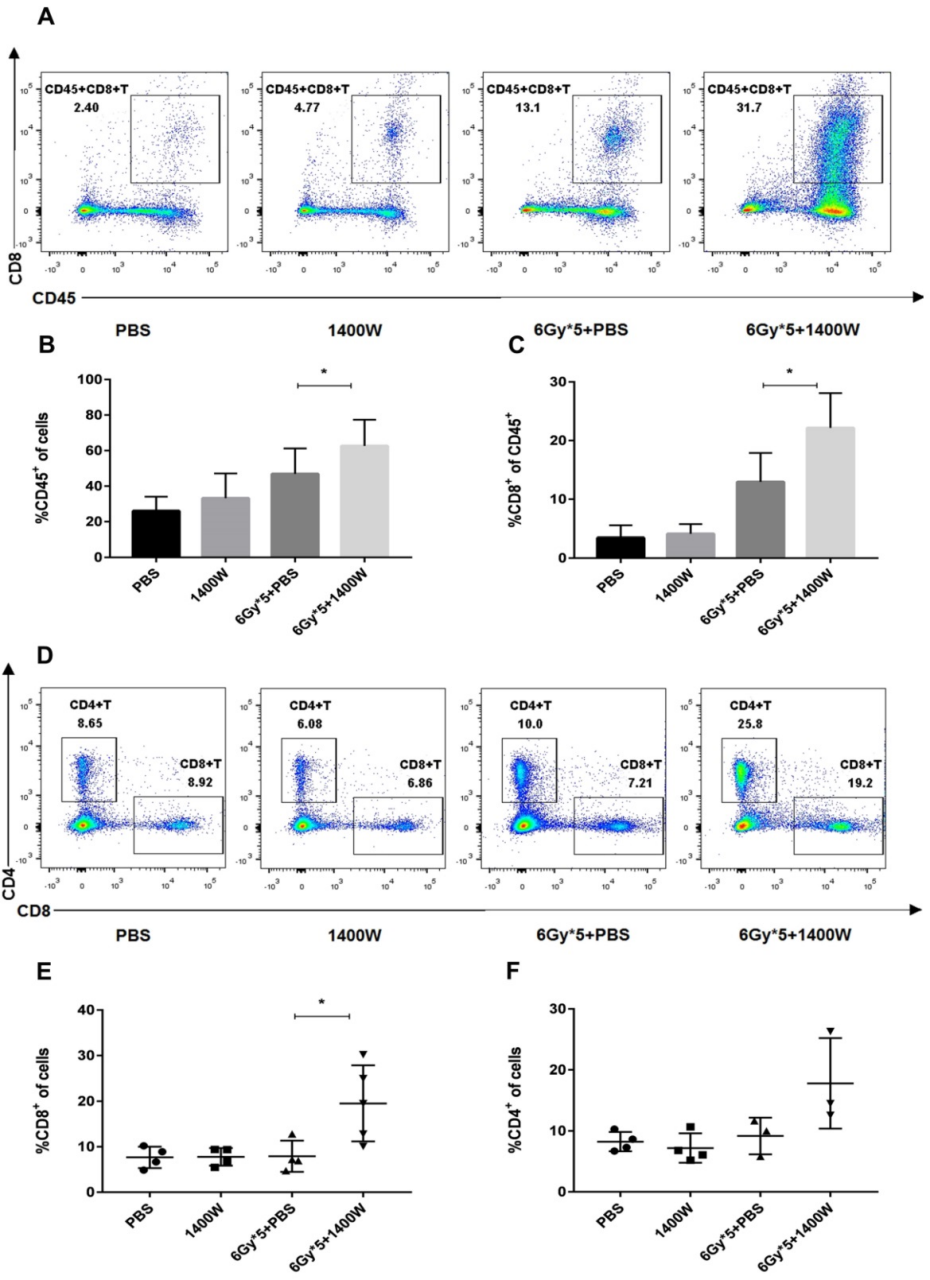
**iNOS inhibition combined with RT to alter the levels of tumor- and spleen-infiltrating T lymphocytes.** Tumor tissues were excised and serum samples were collected from peripheral blood 5 days after RT. (A): Representative cytometry of tumor-infiltrating CD45^+^ and CD8^+^ lymphocytes in the lung cancer tissues. n ≥ 3; *, P < 0.05. (B): Quantitative analysis of tumor-infiltrating CD45^+^ T cells in the lung cancer tissues. n ≥ 3; *, P < 0.05. (C): Quantitative analysis of tumor-infiltrating CD45^+^CD8^+^ T cells in the lung cancer tissues. n ≥ 3; *, P < 0.05. (D): Representative cytometry of CD8^+^ and CD4^+^ T cells in the spleen. n ≥ 3; *, P < 0.05. (E): Quantitative analysis of CD8^+^ T cells in the spleen. n ≥ 3; *, P < 0.05. (F): Quantitative analysis of CD4^+^ T cells in the spleen. n ≥ 3; *, *P* < 0.05.

**Figure 4:**
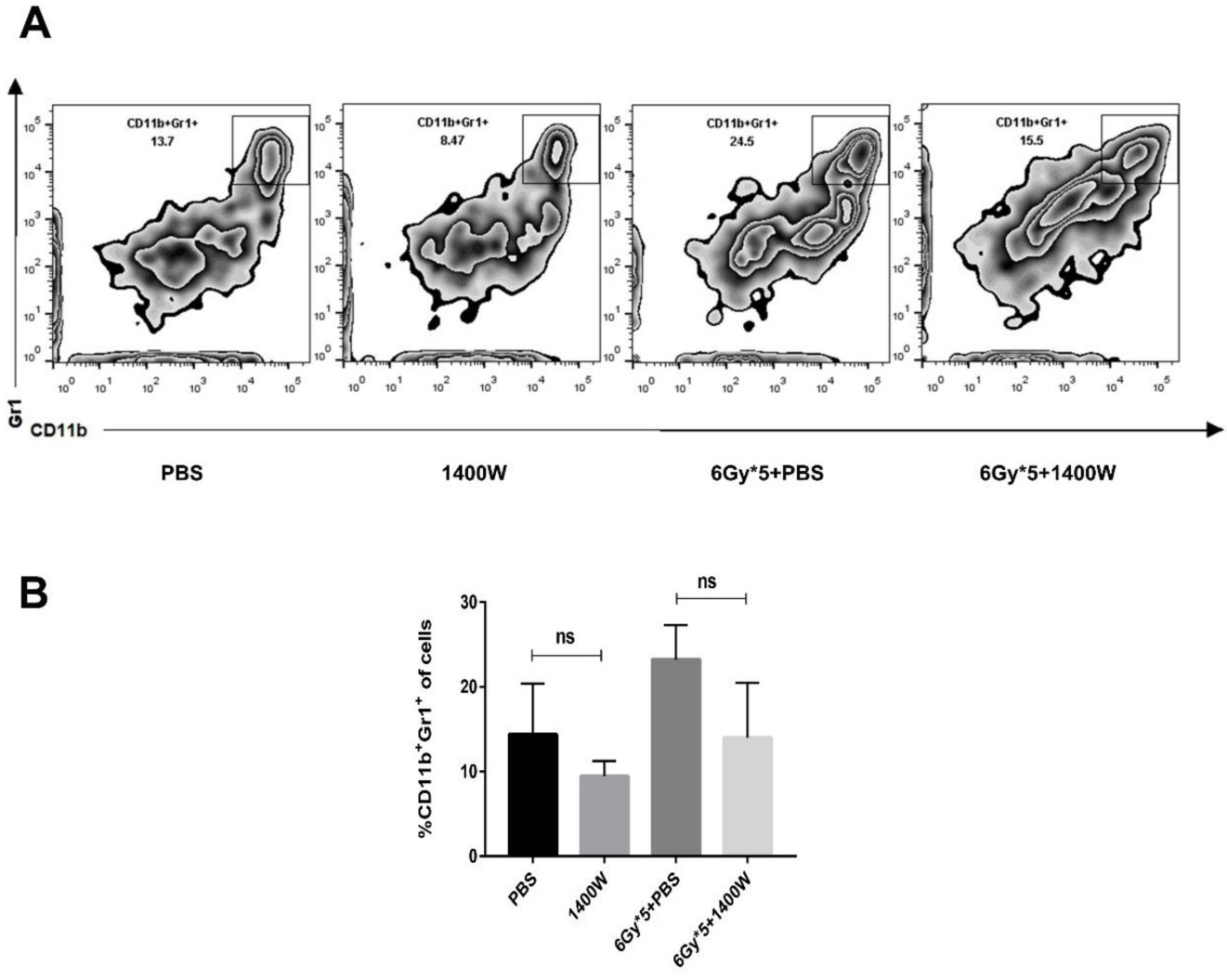
** iNOS inhibition had no effect on the levels of tumor-infiltrating myeloid-derived suppressor cells.** (A): Representative cytometry of tumor-infiltrating MDSCs in lung cancer tissues. (B): Quantitative analysis of MDSCs in lung cancer tissues. n ≥ 3; ns, not significant.

Myeloid-derived suppressive cells inhibit T cell proliferation and function, suppressing immune system activation, and iNOS is involved in the regulation of this process. Based on the results above, the possible relationship between tumor growth delay and activation of the immune system was further investigated. Tumor-bearing C57BL/6 mice were sacrificed 5 days after irradiation treatment. Tumor tissues and spleens were excised for T lymphocyte analysis. After tumor digestion with collagenase, single-cell suspensions were stained with fluorescence-labeled CD45, CD8, and CD4 antibodies. The results indicated that the percentage of tumor-infiltrating CD45^+^ cells was collaboratively upregulated by iNOS inhibition and RT (Fig. 3A&B, *P* < 0.05). iNOS inhibition combined with RT also markedly increased the percentage of CD45^+^ CD8^+^ T cells in lung cancer tissues (Fig. 3C; *P* < 0.05). Examination of CD8^+^ and CD4^+^ T cells in the spleen also showed that the group receiving combined treatment had a notable increase in the percentage of CD8^+^ and CD4^+^ T cells compared with single treatment groups (Fig. 3D-F). MDSCs in the tumor microenvironment were also detected by flow cytometry, but no statistically significant differences were found between the groups (Fig. 4).

**Figure 5:**
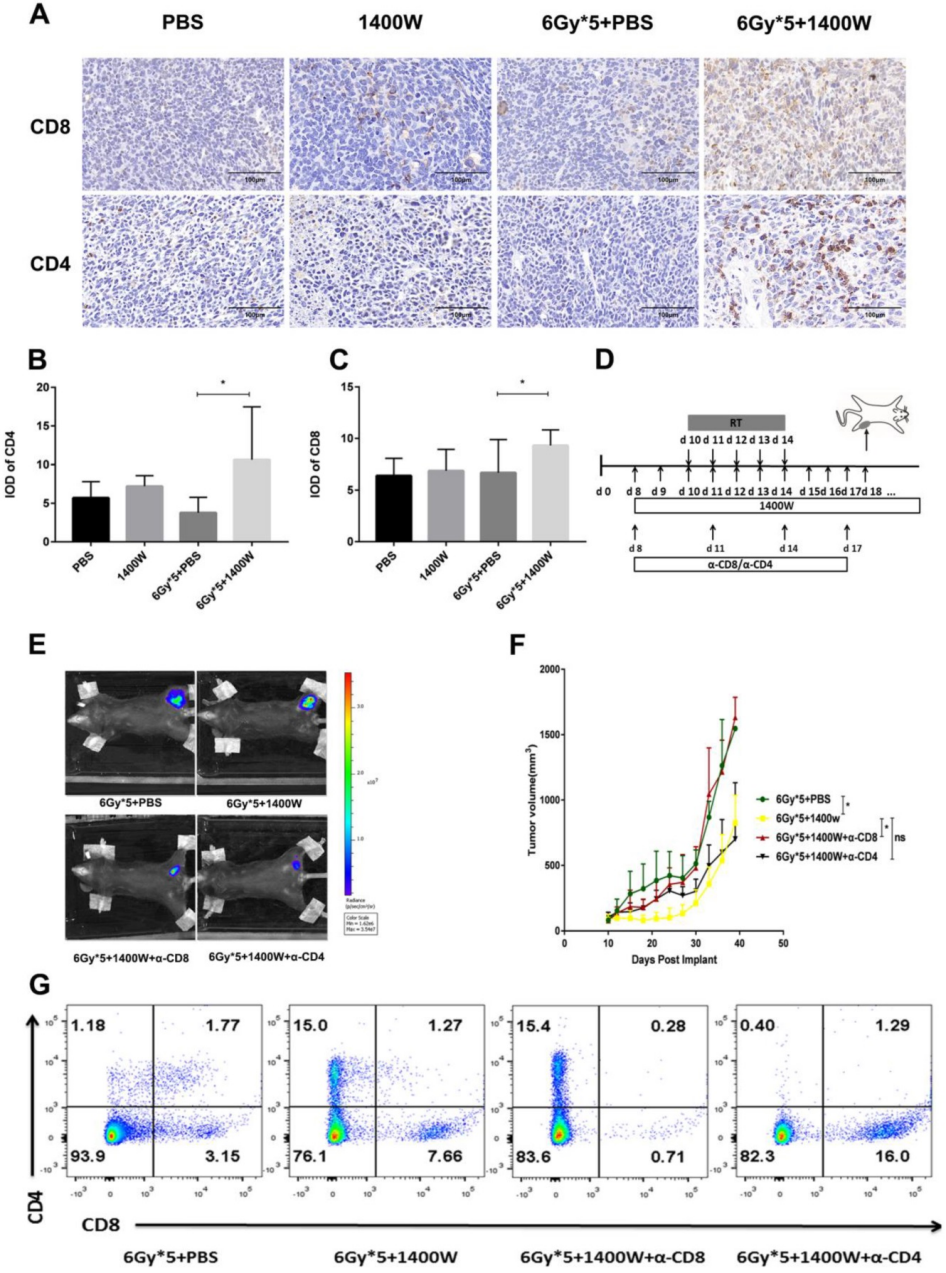
** CD8+ T cells mediated the increased efficacy of RT by iNOS inhibition.** (A): Representative immunohistochemistry images of CD4^+^ and CD8^+^ T cells in lung cancer tissues. (B-C): Quantification of CD4^+^ and CD8^+^ T cells in lung cancer tissues. *, P < 0.05. (D): Treatment schema for CD8^+^ and CD4^+^ T cell depletion study. (E): Representative *in vivo* images of mice with CD8^+^ or CD4^+^ T cell depletion. (F): Tumor growth curves of mice with CD8+ or CD4+ T cell depletion. *, P < 0.05. (G): Analysis of peripheral blood lymphocytes to verify the depletion of CD8^+^ or CD4^+^ T cells.

In addition to flow cytometry, immunohistochemistry also showed that iNOS inhibition combined with radiation significantly increased CD4^+^ and CD8^+^ T cells in lung cancer tissues (Fig. 5A-C). A CD4^+^/CD8^+^ T cell depletion experiment was used to further confirm these results (Fig. 5D). Depletion of CD8^+^ T cells effectively abrogated the antitumor effects of the combined therapy. However, a delay in tumor growth with combined treatment was still observed after CD4^+^ T cell depletion (Fig. 5E&F). Successful depletion was verified in peripheral blood (Fig. 5G).

**Figure 6:**
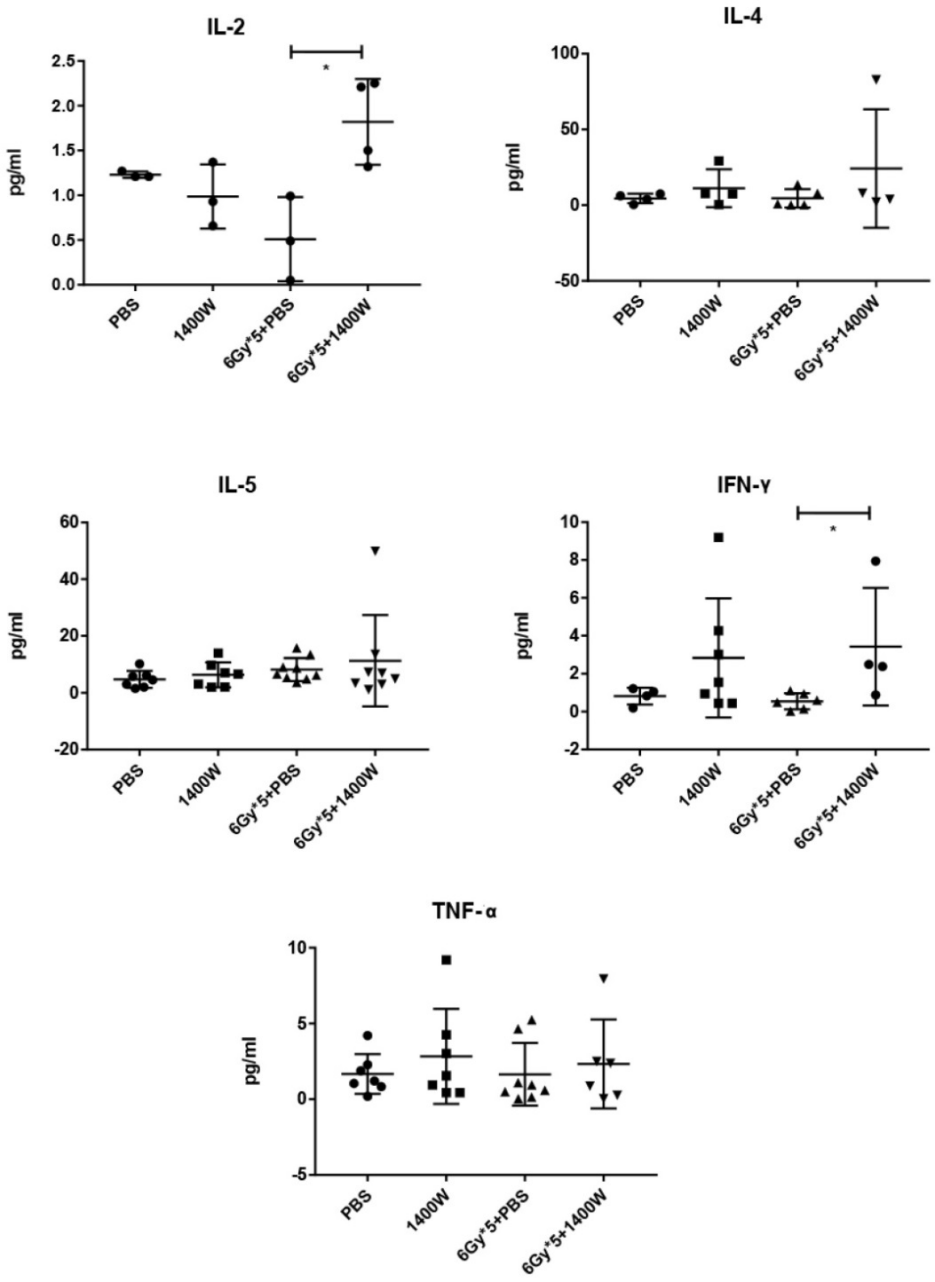
** The combination of iNOS inhibition and RT increased IL-2 amd IFN-γ levels in the serum.** The serum levels of IL-4, IL-5 and TNF-α were comparable between groups. n ≥ 3; *, P < 0.05.

**Figure 7:**
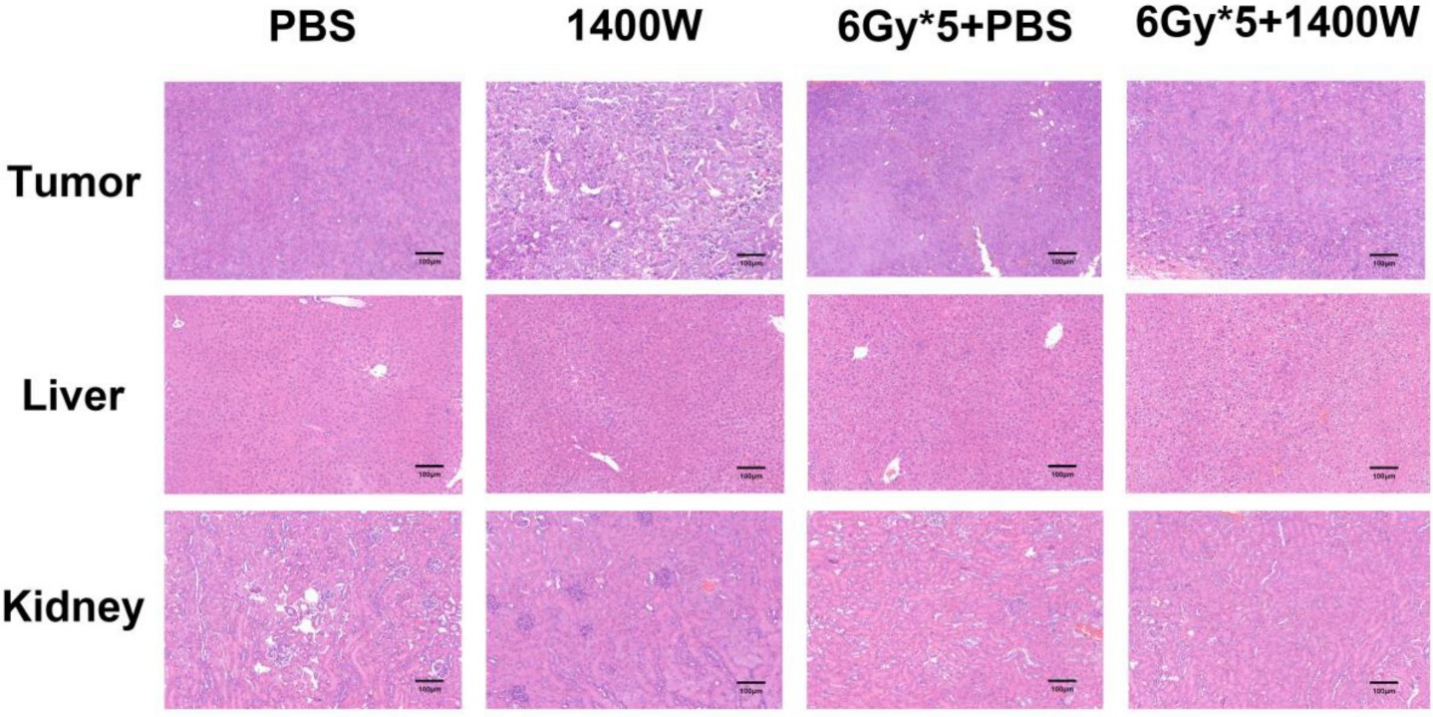
** Representative hematoxylin and eosin staining images of lung cancer tissues, liver and kidney.** No obvious organ toxicity was induced by the combination of iNOS inhibition and RT.

To investigate the serum levels of IL-2, IL-4, IL-5, and IFN-γ, a CBA Mouse Th1/Th2 Cytokine Kit was used. A statistically significant upregulation of IL-2 and IFN-γ was seen in serum collected from the peripheral blood of tumor-bearing mice treated with 1400W and RT (Fig. 6). Hematoxylin and eosin staining of liver and kidney tissues indicated that 1400W had no obvious organ toxicity (Fig. 7).

These results suggested that iNOS inhibition markedly improved the therapeutic effects of RT by upregulating T cell proliferation, thus enhancing the activation of the immune system.

### iNOS inhibition neutralized the myeloid cell-induced inhibition of T cell proliferation *in vitro*

**Figure 8:**
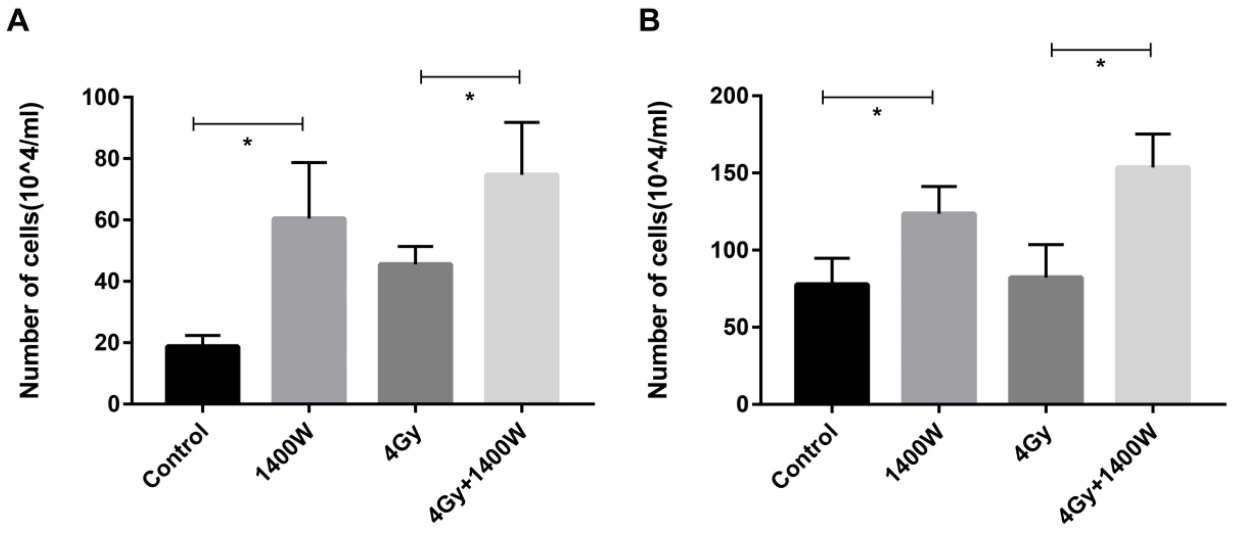
** iNOS inhibition neutralized myeloid cell-induced inhibition of T cell proliferation *in vitro.*** The supernatant was collected in the THP-1 cell culture 12 h (A) and 24 h (B) after irradiation and applied to Jurkat cells. The number of viable Jurkat cells was examined using blood cell counting plate 24 h later. n ≥ 3; *, P < 0.05.

To determine whether the myeloid cell-induced inhibition of T cell proliferation could be counteracted by iNOS inhibition, a cell co-culture experiment was designed. After irradiated THP-1 cells were incubated for 12 h and 24 h (Fig. 8), the supernatant was collected and applied to Jurkat cells. Jurkat cells were incubated for 24 h, after which the number of viable cells was calculated using a blood cell counting plate (Fig. 8A&B). These data indicated a statistically significant increase in the number of T cells in the group treated with 1400W alone, compared with the control group. iNOS inhibition combined with RT also resulted in increased T cell proliferation compared to RT alone. Thus, we concluded that 1400W counteracted the myeloid cell-induced inhibition of T cell proliferation *in vitro*.

## Discussion

Advances in immunotherapy drew increasing attention of cancer treatment recently. In particular, the combination of immunotherapy and traditional RT had substantial progress to improve clinical outcomes 27, 28. RT was reported to activate immune system and prime T cells to tumor antigens 29, 30. However, immunosuppressive myeloid-derived cells, recruited by ionizing radiation, inhibited the activation and proliferation of T cells by producing NO, catalyzed by iNOS 2-4, 31, 32.

Therefore, we reasoned that increased iNOS levels would be immunosuppressive in the tumor environment for the following reasons. Firstly, NO production suppresses the T cell function through divergent mechanisms that involve reversibly blocking signaling through the IL-2 receptor, inhibiting the expression of MHC class II molecules, suppressing the function of JAK3 and STAT5 in T cells, and mediating the apoptosis of T cells 2-4, 33, 34. Secondly, L-arginine is indispensable for the proliferation of cytotoxic lymphocytes, but it can be depleted by iNOS 16-18, 35-38. Lastly, iNOS is over-expressed in many cancers, including melanoma and gastric, breast, colon, and head and neck carcinomas 39-43. Additionally, clinical data shows that iNOS levels may be a predictor of poor survival 44.

We previously reported that RT-induced T cell initiation can be inhibited by iNOS 12. In this study, we showed that blocking iNOS in combination with RT effectively slowed tumor growth and prolonged survival in a mice model. It has been reported that RT alone can recruit CD8^+^ T cells to irradiated tumors 30, 45, 46. Our current data also showed that RT alone primed CD8^+^ T cells in established tumors. However, combined therapy resulted in a more pronounced increase in CD8^+^ T cell infiltration.

The CD4^+^ T cell population is composed of several subgroups, such as Th1, Th2, and Tregs, of which Th2 CD4^+^ T cells and Tregs act as immunosuppressive cells. Thus, there is skepticism regarding the anti-tumor effects of CD4^+^ T cells. In our study, CD4^+^ T cells were not as essential as CD8^+^ T cells for the therapeutic effect of RT and 1400W combination treatment. The depletion of CD8^+^ T cells completely abrogated the suppression of the irradiated tumor. However, CD4^+^ T cell depletion had minimal effect, which demonstrated the indispensable role of CD8^+^ T cells.

Tumor-infiltrating myeloid-derived cells are mainly composed of dendritic cells, MDSCs, polymorphonuclear neutrophils, and TAMs. In our co-culture experiments *in vitro*, we selected THP-1 cells, which could differentiate into immunosuppressive macrophages. We verified the interaction between these macrophages and T cells. Interestingly, flow cytometry showed no effect of iNOS inhibition on the levels of tumor-infiltrating myeloid-derived suppressor cells. The inhibition of iNOS might be more involved in the interaction between myeloid- derived cells and T cells, rather than directly acting on the former.

To investigate the mechanism through which local RT influenced the anti-tumor immune response, we examined the serum levels of cytokines relevant to inflammation. Previous studies showed that IFN-γ and IL-2 appeared to be immune-promoting. IFN-γ had a tumor cell-killing effect 47, while IL-2 was involved in facilitating CD8+ T cell differentiation and activation 48. IL-4 and IL-5 were known as immunosuppressive cytokines 49. Our data showed that combined treatment increased the serum levels of IFN-γ and IL-2, which indicated a synergistic antitumor effect of RT and 1400W. Interestingly, our data showed no differences among groups of the other three cytokines, IL-4, IL-5 and TNF-α, which were pro-inflammatory cytokines.

RT can induce the immunogenic death of tumor cells and the subsequent generation of DAMPs contributes to the efficacy of radiation-induced *in situ* vaccines 50. After treatment with a combination of RT and 1400W, both the increase in CD8^+^ T cell infiltration in the spleen and the up-regulation of certain inflammatory cytokines in serum, showed that this treatment was effective at activating systemic immunity. It has been reported that NO is the agent produced by TAMs that can directly interfere with T cell activation and proliferation 2-4, 31, 32. Our *in vitro* data also demonstrated that the addition of an iNOS inhibitor abrogated the suppression of T cell proliferation by irradiated THP-1-derived macrophages.

## Conclusions

In this study, our *in vivo* assays demonstrated that iNOS inhibition combined with RT led to an apparent increase in tumor- and spleen-infiltrating CD8^+^ T cells and upregulated serum inflammatory cytokine levels. Moreover, our *in vitro* experiments indicated that iNOS blockade neutralized the inhibitory effects of myeloid cells on T cell proliferation. The mechanism whereby iNOS inhibition sensitizes RT was thus revealed. Our findings supported the potential of 1400W as a radiation sensitizer for application in combined therapies to treat various solid tumors.

## Abbreviations

NO: nitric oxide
iNOS: inducible NO synthase
RT: radiotherapy
MDSCs: myeloid-derived suppressor cells
TAM: tumor-associated macrophage
LLC: Lewis lung carcinoma
PBS: phosphate-buffered saline
IL: interleukin
IFN: interferon
CBA: Cytometric Bead Array
